# Long-Term Effect of Elevated CO_2_ on the Development and Nutrition Contents of the Pea Aphid (*Acyrthosiphon pisum*)

**DOI:** 10.3389/fphys.2021.688220

**Published:** 2021-06-04

**Authors:** Chunchun Li, Qian Sun, Yuping Gou, Kexin Zhang, Qiangyan Zhang, Jing-Jiang Zhou, Changzhong Liu

**Affiliations:** ^1^College of Plant Protection, Gansu Agricultural University, Lanzhou, China; ^2^State Key Laboratory of Green Pesticide and Agricultural Bioengineering, Ministry of Education, Guizhou University, Guiyang, China

**Keywords:** *Acyrthosiphon pisum*, elevated CO_2_, generation, development, nutrition

## Abstract

It is predicted that the current atmospheric CO_2_ level will be doubled by the end of this century. Here, we investigate the impacts of elevated CO_2_ (550 and 750 μL/L) on the development and nutrition status of the green pea aphid for six generations, which is longer than previous studies. All seven examined physiological parameters were not affected over six generations under the ambient CO_2_ level (380 μL/L). However, the elevated CO_2_ levels (550 and 750 μL/L) prolonged nymph duration, decreased adult longevity, female fecundity and protein content, and increased the contents of total lipid, soluble sugar and glycogen. There was a significant interaction between the effect of CO_2_ levels and the effect of generations on nymph duration, female fecundity and adult longevity. The elevated CO_2_ had immediate effects on the female fecundity and the contents of total protein, total lipid and soluble sugar, starting within F_0_ generation. The adult longevity decreased, and the glycogen content increased from the F_1_ generation. However, the significant effect on the nymph development was only observed after three generations. Our study indicates that the elevated CO_2_ levels first influence the reproduction, the nutrition and the energy supply, then initiate aphid emergency responses by shortening lifespan and increasing glucose metabolism, and finally result in the slow development under further persistent elevated CO_2_ conditions after three generations, possibly leading to population decline under elevated CO_2_ conditions. Our results will guide further field experiments under climate change conditions to evaluate the effects of elevated CO_2_ on the development of the pea aphids and other insects, and to predict the population dynamics of the green pea aphid.

## Introduction

A rise in atmospheric carbon dioxide (CO_2_) concentration is the most conspicuous characteristics of global climate change in this century (Michael, 2019). CO_2_ concentration is predicted to continue to increase from the current 400 ppm to between 750 and 1,300 ppm by the end of this century (IPCC, 2014). The elevated CO_2_ will not only accelerate the process of global warming, sea level rise, and climate anomalies, but also affect the survival and distribution of plants and animals on the earth, thus having a profound impact on the entire ecosystem (Bezemer and Jones, 1998; Dury et al., 1998; Guo et al., 2013a).

The effects of elevated atmospheric CO_2_ concentration on plant nutritional and defensive chemistry, and their consequent effects on insect have received extensive attention (DeLucia et al., 2012; Facey et al., 2014). In particular, unprecedented increases in atmospheric CO_2_ have the capacity to change plant chemistry, thus, impacting plant nutrients and leading to changes in the synergistic relationship between insects and plants (Robinson et al., 2012; Zavala et al., 2013; Ode et al., 2014). For example, the effect of elevated CO_2_ on the physiological metabolism of aphid host plant alfalfa *(Medicago sativa* L.*)* has been shown previously (Sun et al., 2019), which discovered that the concentrations of soluble protein, soluble carbohydrate and starch were higher when plants grown under elevated CO_2_, also the concentration of flavone, total polyphenols and simple phenols increased significantly in alfalfa. The concentrations of phenolics, terpenoids, condense tannins, and gossypol were increased in *Gossypium hirsutum* by elevated CO_2_ (Coviella et al., 2002). The elevated CO_2_ mediated the decrease of plant N content resulting in a nutritional deficiency for protein-limited insect pests (Mattson, 1980; Coviella and Trumble, 1999) and reduced fecundity and fitness of most leaf-chewing insects (Coll and Hughes, 2008). The CO_2_-mediated lower nitrogen content and higher C: N ratio and thus a decrease nutritional quality of *Zea mays* caused a significant decline in the survival and weight gain as well as larval food consumption of *Ostrinia furnacalis* (Xie et al., 2015). The growth of *Lymantria dispar* larvae was significantly inhibited by elevated CO_2_ and CO_2_-induced changes in quality of leaves of *Populus pseudosimonii* and *Betula platyphylla* (Ji et al., 2011). Similarly, the population density and body mass of the vine weevils (*Otiorhynchus sulcatus*) decreased under elevated CO_2_ (Johnson et al., 2011). Johnson et al. (2020) reported that the elevated CO_2_ accelerated the growth rates of the cotton bollworm (*Helicoverpa armigera*). Furthermore, insect responses to rising CO_2_ are not uniform. Robinson et al. (2012) found that while Lepidoptera populations decreased with elevated CO_2_, the populations of other groups such as Homoptera and Acari actually increased.

Wu et al. (2006) found that the elevated CO_2_ concentration (750 μL/L) decreased the protein and total amino acid contents of *Helicoverpa armigera*. Similarly, the study on the larvae of *Stegobium paniceum* and *Lasioderma serricorne* showed that the contents of polysaccharide, soluble protein and lipids in the larvae as well as the utilization rate of the larvae decreased with increasing CO_2_ concentration (Cao et al., 2006). Some studies suggested that the elevated CO_2_ concentration can increase the relative feeding rate, prolong the development time, decrease the relative growth rate and pupa weight in chewing mouthparts insects (Bidart-Bouzat and Imeth-Nathaniel, 2008; Massad and Dyer, 2010). For example, the larva-to-adult emergence survival rate of *Cnaphalocrocis medinalis* decreased by 44.0%, and the egg hatching rate reduced by 26.8% under the elevated CO_2_ as compared to the ambient CO_2_ (Li et al., 2013).

Aphids are considered to be the most important group of insect pests in temperate regions because of its rapid parthenogenic reproduction and short life cycle. Hughes and Bazzaz (2001) investigated interactions between five species of phloem-feeding aphids (Homoptera: Aphididae) at elevated CO_2_ (700 μL/L) compared to the ambient CO_2_ (350 μL/L), and found that *Acyrthosiphon pisum* population were reduced by over 60% at elevated CO_2_, in contrast, *Myzus persicae* populations increased by 120% at elevated CO_2_, but the CO_2_ treatment did not significantly affect the populations of the remaining three species (*Aphis nerii*, *Aphis oenotherae*, *Aulacorthum solani*). Also, under elevated CO_2_, winged aphid adults were produced in all the aphid species except *Myzus persicae*, but the proportion of winged to wingless was not affected by CO_2_ levels. The pea aphid, *A. pisum* Harris, is a worldwide pest that feeds on leguminous crops and forages grasses such as peas, dry beans, alfalfa, clover and fresh beans (Harrington, 1945). Its rapid parthenogenic reproduction and short life cycle enable them to very quickly cause serious economic and production losses of host crops (Losey and Eubanks, 2000; Ryalls et al., 2013). In addition to the direct feeding damage, the pea aphids also transmit 25 types of viruses, including *Alfalfa mosaic virus* and *Pea enation mosaicvirus* (Makkouk, 1988). In previous studies, many factors that affect the growth and development of the pea aphid were investigated from different perspectives, including temperature (Hubhachen et al., 2018), photoperiod (Miquel et al., 2019), symbiont (Lv et al., 2018), genetics (Caillaud and Losey, 2010; Wang et al., 2012), and host plant quality (Kordan et al., 2018). Among them rising global CO_2_ concentrations has become an important climatic factor affecting pea aphid growth, for example, elevated CO_2_ concentrations altered plant phenolics and thus the performance of aphids (Hong et al., 2020). Studies found that the aphids grown at 380 ppm CO_2_ had the longest pre-reproductive period and the aphids grown at 1,050 ppm CO_2_ had the highest intrinsic rate of natural increase (Amiri-jami et al., 2012). Li and Liu (2017) found that developmental duration of three consecutive generations of green pea aphids was shortened, while larval weight, adult weight, weight difference and mean daily relative growth rate all displayed increasing trends under elevated (750 μL/L) CO_2_. However, the biochemistry and genetic bases of the long-term effect of elevated CO_2_ on aphid development and nutrients are not investigated.

The objectives of this study are to analyze the independent and interactive effects of two elevated CO_2_ levels on the development and nutrition content of the pea aphids over six generations. The aim of this study is to provide new and comprehensive insights into the function of CO_2_ as well as its potential role in control strategies of pea aphids in agricultural systems.

## Materials and Methods

### Insects and CO_2_ Treatment

The stock colony of the green pea aphid *Acyrthosiphon pisum* Harris was from a single parthenogenetic female collected from the alfalfa (*Medicago sativa* L) field in Experimental Station of the Gansu Agricultural University, Lanzhou, Gansu Province, China. Fresh and clean alfalfa leaves were placed on the filter paper in 9 cm petri dishes with the back facing up and moist absorbent cotton around the petioles and leaf edges to ensure the leaves fresh. Several small holes were drilled on the lid of the petri dish to facilitate the passage of CO_2_. Three levels of CO_2_, i.e., ambient CO_2_ (∼380 μL/L) and elevated CO_2_ (550 and 750 μL/L), were applied continuously to the petri dishes with the aphids in separate rearing chambers. The CO_2_ levels were chosen based on the current and predicted levels of CO_2_ in future years, respectively (Watson et al., 1996).

### Determination of Aphid Development for Six Generations

Twenty newly emerged (<6 h) nymph from the aphid stocks were individually placed into a previously prepared petri dish and treated with one of the three CO_2_ levels for more than 30 days in a rearing chamber. The broad alfalfa leaves were changed every 3 days during the experiment. The observations were made daily at 8 h intervals until the adults died. Then another 20 nymphs (<6 h) in the petri dishes were individually transferred to new petri dishes with fresh leaves and kept for next run of the treatments and observations. This procedure was repeated six times thus six generations (F_0_–F_5_) were examined under each CO_2_ level. This made 20 replicates for each treatment at each generation. Each treatment was repeated three times for each CO_2_ level. A total of 1,080 nymphs (20 nymphs × 3 treatments × 6 generations × 3 replicates) was used.

The duration of each nymphal stage and the molting during the nymph stages in each petri dish were recorded daily at 8 h intervals until the adults died. The number of nymphs produced per aphid and the adult longevity were recorded when the adults died. Nymphal duration was between hatching and adult emergence.

### Sample Preparation for Biochemistry Measurements

A total of 135 adult aphids was collected from each generation for the detections of total protein, soluble sugar, glycogen and lipid under the influence of CO_2_. Briefly, five adult aphids from each generation were placed into a 1.5 mL centrifuge tube containing 200 μL of the lysis buffer solution (100 mM KH_2_PO_4_, 1 mM ethylenediaminetetraacetic acid, and 1 mM dithiothreitol, pH 7.4). The aphids were homogenized and then centrifuged at 10,000 rpm for 10 min at 4°C. The supernatant was then used as one biological sample for the nutritional detections of total protein, sugar, glycogen and lipid. 20 μL of the biological sample was used for the protein content measurement and another 160 μL was used for the measurements of lipids, soluble sugar and glycogen content. This biological sample preparation of biochemistry measurements was repeated for nine times for each of three CO_2_ treatments (380, 550, and 750 μL/L).

### Determination of Total Protein Content

The total protein content of the aphids was detected according to the method of Lowry et al. (1951). About 20 μL of the biological sample was transferred into a 96-well microplate and mixed with of 200 μL Coomassie brilliant blue G-250 for 15 min at room temperature. The optical density (OD) values were measured at 595 nm. Bovine serum albumin was dissolved in the same buffer and diluted into a series of concentrations, which was used as the standard. The total protein contents were calculated based on the standard curve of bovine serum albumin. The experiment was repeated nine times.

### Sample Preparation for the Measurements of Total Lipid, Soluble Sugar, and Glycogen Content

Briefly, 160 μL of the biological sample was transferred into a 2 mL centrifuge tube, and 20 μL of 20% sodium sulfate solution (Na_2_SO_4_; Sigma) was added, then mixed with 1,500 μL of a chloroform–methanol solution (1:2v/v). This mixture was then centrifuged at 10,000 rpm for 15 min at 4°C. The supernatant was used for the nutritional detection of lipids and soluble sugar, and the precipitant was used for the glycogen content measurement. The preparation was prepared nine times for each CO_2_ treatment.

### Determination of Total Lipids Content

The total lipid content was detected according to the methods of Handel (1985). Briefly, about 100 μL of the supernatant was transferred into a 1 mL centrifuge tube and heated at 90°C until solvent was completely evaporated. 10 μL of 98% concentrated sulfuric acid was then added to the tube and incubated at 90°C for 2 min in a water bath. After cooling the samples on ice, 190 μL of 1.2 g/L vanillin reagent was added and reacted for 15 min at room temperature. The OD value was measured at 525 nm, and the total lipid content was calculated based on the standard curve of triolein. The experiment was repeated nine times.

### Determination of Soluble Sugar

The soluble sugar and glycogen were determined according to the methods of Handel (1965) and Handel and Day (1988). To detect the soluble sugar, 150 μL of the supernatant was transferred into a 1.5 mL centrifuge tube and completely evaporated it at room temperature. 10 μL of distilled water and 240 μL of anthrone reagent were added, kept for 15 min at room temperature and then incubated in boiling water for a further 15 min, followed by cooling at room temperature, then added to a 96-well microplate. The OD value at the 630 nm wavelength was measured. The soluble sugar content was calculated based on the standard curve of D-glucose. The experiment was repeated nine times.

### Determination of Glycogen Content

The precipitant from pre-preparation was mixed with 400 μL of 80% methanol and sonicated for 10 min to make it turbid. The resultant homogenate was centrifuged again at 10,000 rpm for 4 min at 4°C. Next, the supernatant was transferred to a new 2 mL centrifuge tube and mixed with 1,200 μL of anthrone reagent and incubated for 15 min at room temperature. The samples were heated for 15 min at 90°C in a water bath. The reaction was stopped by cooling with cold water. Finally, the absorbance value of the samples was read at 630 nm and the glycogen content was calculated using D-glucose as standard. The experiment was repeated nine times.

### Statistical Analyses

All data were analyzed with the IBM SPSS Statistics version 23.0 for Windows (Chicago, IL, United States). A two-way ANOVA was conducted to examine the effects of CO_2_ level, generation and the interaction between the CO_2_ level and the generation on the physiological parameters (nymphal duration, adult longevity, female fecundity and nutrition contents) followed by Tukey’s HSD test was applied to determine differences specific treatments. The general linear model (GML) procedure of SAS was used to compare the means there is a significant interaction effects between generation and CO_2_ level is identified. Duncan’s Multiple Range Test to compare the means of the physiological parameters if the interaction effect is not significant.

## Results

### There Is a Significant Interaction Between CO_2_ Levels and Generation

There was a statistically significant interaction between the effects of CO_2_ level and generation on the nymph duration (*F* = 36.3; *df* = 10; *p <* 0.001; Table 1). This indicates that the effect CO_2_ levels on nymph duration is dependent on the generation, and the nymph duration at each generation is dependent on the CO_2_ levels. Similarly, significant interactions were found for the measurements of adult longevity (*F* = 13.1; *df* = 10; *p <* 0.001), female fecundity (*F* = 17.3; *df* = 10; *p <* 0.001), protein content (*F* = 20.3; *df* = 10; *p* < 0.001) and sugar content (*F* = 9.5; *df* = 10; *p <* 0.001; Table 1). So, to interpret the effect of CO_2_ levels and generations without inference of each other, one-way ANOVA followed by Tukey’s HSD test was used to separately analyze the effect of CO_2_ level at each generation and the effect of generation under each CO_2_ level on each of the performance indices.

**TABLE 1:** Two factors ANOVA analysis of the effect of CO_2_ *generation interaction on performance indices in *Acyrthosiphon pisum.*

Measurement	Type III sun of squares	df	Mean square	*F*-value	*p*-value
Nymph duration	42.4	10	4.2	36.3	0.000
Adult longevity	80.9	10	8.1	13.1	0.000
Fecundity	2,322.6	10	232.3	17.3	0.000
Protein	134.5	10	13.5	20.3	0.000
Total lipid	2.3	10	0.2	0.6	0.787
Soluble sugar	72.5	10	7.4	9.5	0.000
Glycogen	1.1	10	0.1	1.2	0.308

### *A. pisum* Nymph Duration Is Increased by Elevated CO_2_

**FIGURE 1:**
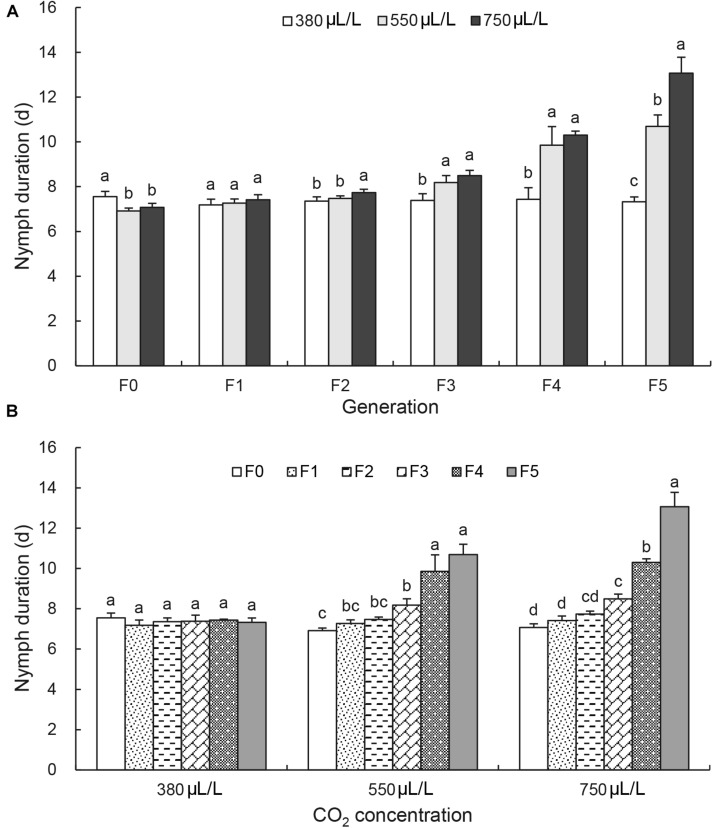
Effects of CO_2_ and generations on the nymph duration of A*cyrthosiphon pisum*. **(A)** Comparison of the effect of CO_2_ levels (380, 550, and 750 μL/L) at each of six generations (F_0_–F_5_). **(B)** Comparison of the effect of generations under each of CO_2_ levels (380 μL/L, elevated 550 and 750 μL/L). Different lowercase letters indicate significant differences between CO_2_ levels at each generation **(A)** and between generations at each CO_2_ level **(B)** as determined by one-way ANOVA Tukey’s HSD test at *p* < 0.05.

Figure 1A shows the Tukey’s HSD test results of the effect of CO_2_ levels on the nymph duration at each generation. It revealed that the nymph duration was shortened at F_0_ generation by the elevated CO_2_ levels [*F*_(2, 6)_ = 9.5, *p* = 0.014; Figure 1A]. The nymph duration at F_1_ generation was not significantly affected by the elevated CO_2_ levels. It was significantly increased by the elevated CO_2_ levels only after F_3_ generation [*F*_(2, 6)_ = 12.0, *p* = 0.008; Figure 1A]. At F_5_ generation, the nymph duration was increased significantly by the elevated CO_2_ levels from 7.3 ± 0.2 days at 380 μL/L to 10.7 ± 0.5 days at 550 μL/L and 13.1 ± 0.7 days at 750 μL/L [*F*_(2, 6)_ = 91.5, *p* < 0.001; Figure 1A].

Figure 1B shows the Tukey’s HSD test results of the effect of generations on the nymph duration at each CO_2_ level. Under the ambient CO_2_ level 380 μL/L, the nymph duration was not changed and kept a similar length of 7.4 ± 0.3 days throughout 6 generations (Figure 1B). Under elevated CO_2_, the nymph duration had a significant increase at F_4_ and F_5_ generations [*F*_(5, 12)_ = 38.3, under 550 μL/L CO_2_ level, *p* < 0.001; *F*_(5, 12)_ = 135.5 under 750 μL/L CO_2_ level, *p* < 0.001].

### *A. pisum* Adult Longevity Is Decreased by Elevated CO_2_

**FIGURE 2:**
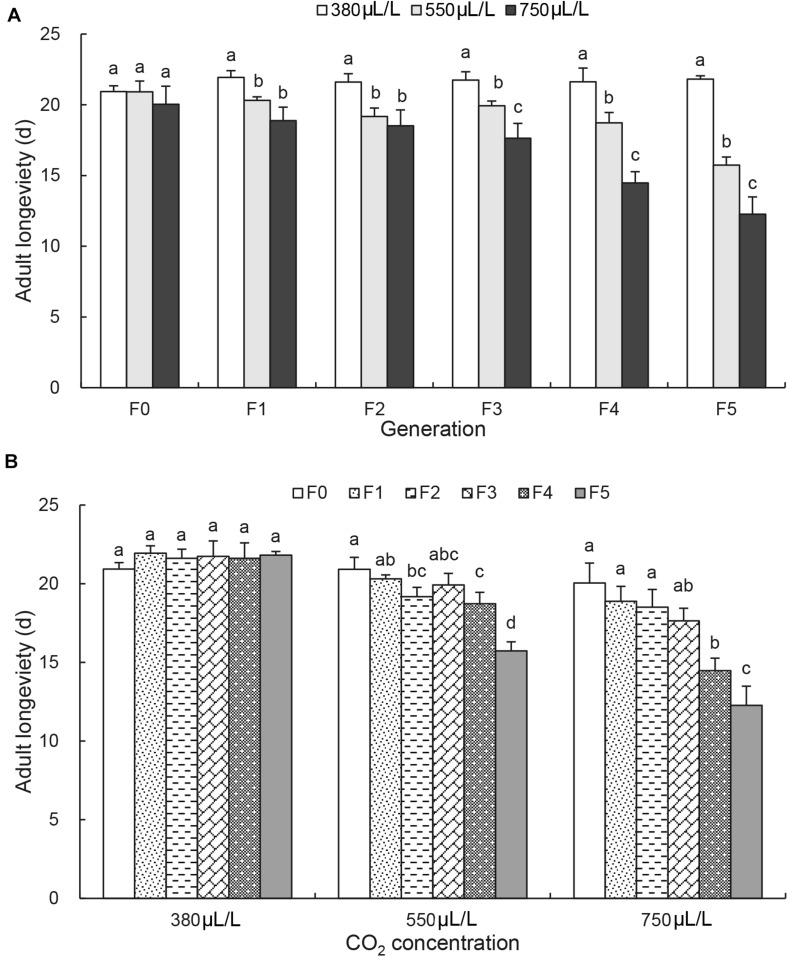
Effects of CO_2_ and generations on the adult longevity of *Acyrthosiphon pisum*. **(A)** Comparison of the effect of CO_2_ levels (380, 550, and 750 μL/L) at each of six generations (F_0_–F_5_). **(B)** Comparison of the effect of generations under each of CO_2_ levels (380 μL/L, elevated 550 and 750 μL/L). Different lowercase letters indicate significant differences between CO_2_ levels at each generation **(A)** and between generations at each CO_2_ level **(B)** as determined by one-way ANOVA Tukey’s HSD test at *p* < 0.05.

The adult longevity was not affected by CO_2_ levels and remained constant of 20.6 ± 0.8 days at F_0_ generation (Figure 2A). It was decreased significantly from F_1_ generation by elevated CO_2_ levels and reduced from 21.8 ± 0.2 days at 380 μL/L to 15.7 ± 0.6 days at 550 μL/L and 12.3 ± 1.2 days at 750 μL/L at F_5_ generation [*F*_(2, 6)_ = 111.3, *p* < 0.001; Figure 2A]. Similarly, the adult longevity also remained constant days under the ambient CO_2_ level (380μL/L) over the generations (Figure 2B). However, it started to decrease significantly under the elevated CO_2_ levels. Under the elevated CO_2_ level of 750 μL/L, the adult longevity was significantly reduced to 12.3 ± 1.2 days by F_5_ generation [*F*_(5, 12)_ = 22.8, *p* < 0.001; Figure 2B].

### *A. pisum* Fecundity Is Significantly Reduced by Elevated CO_2_

**FIGURE 3:**
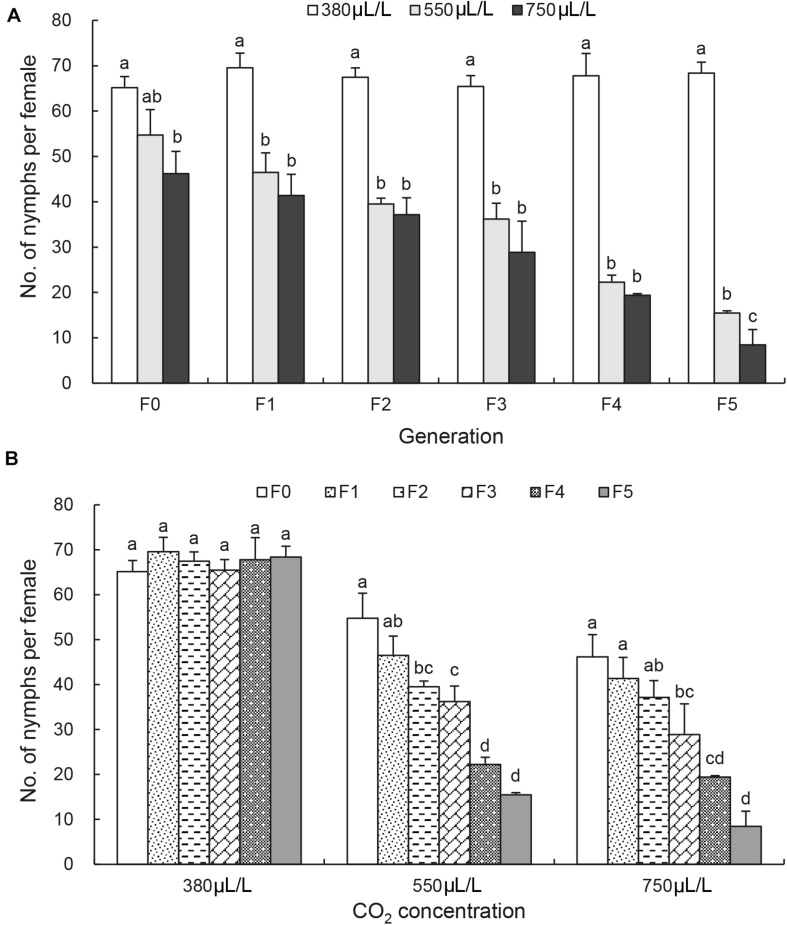
Effects of CO_2_ and generations on the female fecundity of *Acyrthosiphon pisum*. **(A)** Comparison of the effect of CO_2_ levels (380, 550, and 750 μL/L) at each of six generations (F_0_–F_5_). **(B)** Comparison of the effect of generations under each of CO_2_ levels (380 μL/L, elevated 550 and 750 μL/L). Different lowercase letters indicate significant differences between CO_2_ levels at each generation **(A)** and between generations at each CO_2_ level **(B)** as determined by one-way ANOVA Tukey’s HSD test at *p* < 0.05.

At F_0_ generation, the number of nymphs per female was not affected significantly by 550 μL/L CO_2_ level but significantly reduced by 750 μL/L CO_2_ level (Figure 3A). At F_1_ generation, the reduction of the number of nymphs per female became more obvious under the elevated CO_2_ levels (Figure 3A). By F_5_ generation, the number of nymphs per female was reduced significantly from 68.4 ± 2.4 under the ambient CO_2_ level to 15.5 ± 0.5 under 550 μL/L CO_2_ level and to 8.5 ± 3.3 under 750 μL/L CO_2_ level [*F*_(2, 6)_ = 558.2, *p* < 0.001; Figure 3A]. There was no reduction in the number of nymphs per female between generations under the ambient CO_2_ level 380 μL/L (Figure 3B). Significant reduction of the number of nymphs per female was observed between generations under elevated CO_2_ levels [*F*_(5, 12)_ = 58.9 under 550 μL/L CO_2_ level, *p* < 0.001; *F*_(5, 12)_ = 30.9 under 750 μL/L CO_2_ level, *p* < 0.001] when the effect of CO_2_ levels was analyzed (Figure 3B).

### *A. pisum* Total Protein Content Is Reduced Under Elevated CO_2_

The elevated CO_2_ levels significantly reduced the total protein contents even at F_0_ generation [*F*_(2, 24)_ = 127.6, *p* < 0.001; Table 2 and Supplementary Figure 1A]. The total protein content under the ambient CO_2_ was not affected throughout the generations (Table 2 and Supplementary Figure 1B). Under the elevated CO_2_ level of 550μL/L, the total protein contents were significantly reduced from 19.7 ± 0.6 μg/aphid at F_0_ generation to 13.6 ± 0.5 μg/aphid at F_5_ generation [*F*_(5_, _48)_ = 176.9, *p* < 0.001]. Under the elevated CO_2_ level of 750 μL/L, it was reduced from 16.6 ± 0.6 μg/aphid at F_0_ generation to 12.5 ± 0.8 μg/aphid at F_3_ generation and reached 11.4 ± 0.9 μg/aphid at F_5_ generation [*F*_(5, 48)_ = 61.9, *p* < 0.001; Table 2 and Supplementary Figure 1B].

**TABLE 2:** Average content (ug/aphid) of protein, soluble sugar, glycogen and total lipid.

Measurement	CO_2_ levels (μ L/L)	Generation					
		F_0_	F_1_	F_2_	F_3_	F_4_	F_5_
Protein	380	20.9 ± 0.6*Aa*^1^	20.1 ± 1.6^Aa^	20.5 ± 0.6^Aa^	20.6 ± 0.9^Aa^	19.9 ± 1.6^Aa^	19.8 ± 0.5^Aa^
	550	19.7 ± 0.6*B*a	19.5 ± 0.3^Aa^	19.8 ± 0.6^Ba^	18.2 ± 0.6^Bb^	15.9 ± 0.7^Bc^	13.6 ± 0.5^Bd^
	750	16.6 ± 0.6^Ba^	15.2 ± 1.0^Bb^	13.5 ± 0.4^Cc^	12.5 ± 0.8^Cc^	12.6 ± 0.6^Cc^	11.4 ± 0.9^Cd^
Total lipid	380	6.7 ± 1.1^Ba^	6.5 ± 0.4^Ba^	6.4 ± 0.8^Ba^	6.5 ± 0.7^Ba^	6.4 ± 0.3^Ba^	6.4 ± 0.7^Ba^
	550	7.7 ± 0.3^Aa^	7.6 ± 0.6^Aa^	7.6 ± 0.2^Aa^	7.9 ± 0.6^Aa^	7.9 ± 0.4^Aa^	7.9 ± 0.5^Aa^
	750	7.7 ± 0.7^Aa^	7.5 ± 0.2^Aa^	7.5 ± 0.3^Aa^	7.4 ± 0.10^ABa^	7.7 ± 0.3^Aa^	8.0 ± 0.6^Aa^
Soluble sugar	380	25.4 ± 0.6^Ca^	25.0 ± 0.6^Ca^	25.4 ± 1.2^Ba^	25.2 ± 0.8^Ba^	25.8 ± 0.4^Ca^	25.6 ± 0.4^Ca^
	550	33.7 ± 1.0^Bb^	33.9 ± 0.6^Bb^	36.6 ± 0.4^Aa^	36.4 ± 1.6^Aa^	37.1 ± 1.2^Ba^	37.7 ± 0.9^Ba^
	750	36.8 ± 0.5^Ab^	36.7 ± 0.6^Ab^	37.6 ± 1.6^Ab^	37.5 ± 1.0^Ab^	39.6 ± 0.4^Aa^	40.3 ± 0.5^Aa^
Glycogen	380	2.0 ± 0.2^Aa^	2.0 ± 0.1^Ba^	2.0 ± 0.2^Ba^	2.1 ± 0.5^Ba^	2.0 ± 0.1*Ca*	2.0 ± 0.1^Ba^
	550	2.2 ± 0.4^Ab^	2.3 ± 0.5^ABab^	2.3 ± 0.2^ABab^	2.3 ± 0.1^ABab^	2.4 ± 0.3^Bab^	2.6 ± 03ABa
	750	2.3 ± 0.5^Aa^	2.6 ± 0.3^Aa^	2.5 ± 0.3^Aa^	2.6 ± 0.1^Aa^	2.8 ± 0.1^Aa^	2.8 ± 0.1^Aa^

*^1^One-way ANOVA Tukey’s HSD test of the effect of each CO_2_ level between generations (rows) and the effect of each generation between CO_2_ levels (column). Different lowercase letters indicate significant differences (p < 0.05) between generations under each of three CO_2_ levels (rows); different uppercase letters indicate significant differences (p < 0.05) between CO_2_ levels at each of six generation (F_0_–F_5_) (columns).*

### *A. pisum* Total Lipid Content Is Not Affected Under Elevated CO_2_

The total content of total lipids was significantly increased even at F_0_ generation by both elevated CO_2_ levels [*F*_(2, 24)_ = 5.4, *p* = 0.012; Table 2 and Supplementary Figure 2A]. At F_5_ generation, it was increased from 6.4 ± 0.7 μg/aphid under 380 μL/L level to 7.9 ± 0.5 μg/aphid under 550 μL/L level and to 8.0 ± 0.6 μg/aphid under 750 μL/L level [*F*_(2, 24)_ = 17.2, *p* < 0.001]. The total content of lipids was unchanged over the generations under the ambient and elevated CO_2_ levels (Table 2 and Supplementary Figure 2B). The total lipid contents increased by 14.9–23.4% and 13.8–25.0% across generations under the elevated CO_2_ levels of 550 and 750 μL/L, respectively, relative to those under the ambient CO_2_ level.

### *A. pisum* Sugar Content Is Increased Under Elevated CO_2_

The content of soluble sugar and glycogen were significantly increased at each generation (Table 2 and Supplementary Figures 3A, 4A) but were not affected by the elevated CO_2_ levels over generations (Table 2 and Supplementary Figures 3B, 4B). The significant increase was observed at F_0_ generation for the soluble sugar content from 25.4 ± 0.6 μg/aphid at 380 μL/L level to 33.7 ± 1.0 μg/aphid by 550 μL/L level and to 36.8 ± 0.5 μg/aphid by 750 μL/L level [*F*_(2, 24)_ = 557.8, *p* < 0.001]. At F_5_ generation, the soluble sugar was increased from 25.6 ± 0.4 μg/aphid under the ambient CO_2_ to approximately 40.3 ± 0.5 μg/aphid by the elevated CO_2_ 750 μL/L (Table 2).

Under both elevated CO_2_ levels, the content of soluble sugar was slightly and significantly increased after F_2_ generation under 550 μL/L level [*F*_(5, 48)_ = 24.8, *p* < 0.001] and after F_4_ generation under 750 μL/L level [*F*_(5, 48)_ = 26.9, *p* < 0.001; Table 2 and Supplementary Figure 3B].

### *A. pisum* Glycogen Content Is Significantly Increased Under Elevated CO_2_

The glycogen content was significantly increased from F_1_ generation (Table 2 and Supplementary Figure 4A). It was significantly increased from 2.0 ± 0.1 μg/aphid at 380 μL/L level to 2.6 ± 0.3 μg/aphid by 750 μL/L [*F*_(2, 24)_ = 5.7, *p* = 0.010]. At F_5_ generation, the glycogen content increased from 2.0 ± 0.1 μg/aphid under the ambient CO_2_ to approximately 2.8 ± 0.1 μg/aphid by the elevated CO_2_ 750 μL/L (Table 2). The glycogen content was not affected by the elevated CO_2_ levels over generations even under the higher elevated CO_2_ level 750 μL/L (Table 2 and Supplementary Figure 4B).

## Discussion

The current study reports the long-term effects of elevated CO_2_ levels on the development and nutritional dynamics of the pea aphid *A. pisum* over six generations. It confirms that the pea aphid is well adapted to the current environmental CO_2_ level as all seven examined physiological parameters were not affected over 6 generations under the ambient CO_2_ level. However, the elevated CO_2_ levels (550 and 750 μL/L) prolonged the nymph duration (Figure 1), decreased the adult longevity, the female fecundity and the protein content (Figures 2, 3 and Table 2), and increased the contents of total lipid, soluble sugar and glycogen (Table 2).

The elevated CO_2_ had an immediate effect on the female fecundity and the contents of total protein, total lipid and sugar, starting within F_0_ generation. In the current study, only total protein and total lipid contents were analyzed. It is not known whether or not they are influenced by the changes of vitellogenin and triacylglycride. Triacylglycerols is a major lipid in aphid fat body as energy reserves (Ward et al., 1981) and accumulated in aphid fat body under stress (Bergman et al., 1991; Chen et al., 2005). Vitellogenin is a major yolk protein (Arrese and Soulages, 2010) and directly regulates egg maturation and affect ovary development and decrease ovulation (Ge et al., 2019; Huang et al., 2019).

The adult longevity decreased, and the glycogen content increased from F_1_ generation. However, the significant effect on the nymph development was only observed after three generations (Figure 1A). Furthermore, the interactions between the CO_2_ levels and the generations significant influenced nymph duration, female fecundity and adult longevity. The biggest effect of CO_2_ was found on the female fecundity by the generations (Figure 3A), indicating that the elevated CO_2_ may not be conducive to the reproduction of the aphids. The results are consistent with that of the study in *Cnaphalocrocis medinalis*, where elevated CO_2_ reduced the survival rate from larva to adult emergence by 44.0% compared with ambient CO_2_ (Li et al., 2013). Docherty et al. (1997) also reported a significantly reduced fecundity in *Phyllaphis fagi* under the elevated CO_2_ level of 600 μL/L. However, it was reported that elevated CO_2_ levels could increase *Myzus persicae* population (Hughes and Bazzaz, 2001) and *Nilaparvata lugens* population (Xiao et al., 2011). It is possible that the elevated CO_2_ levels first influence the reproduction, the energy supply and the nutrition status, and then initiation of shortening lifespan and increasing glycogen transition to glucoses for emergency escaping, and finally result in the slow development of the pea aphid under persistent elevated CO_2_ conditions, possibly leading to population decline under elevated CO_2_ conditions.

Insects store nutrition in the form of proteins, carbohydrates, glycogen and lipids (Ahsaei et al., 2014). Nutrition storage in insects has significant implications for their survival and reproduction (Makkouk, 1988). Our results show a significant reduction of protein content by the elevated CO_2_ levels (Table 2). This is consistent with previous reports that the fertility of the pea aphid was closely related to the protein contents in the body (Ahsaei et al., 2013). For example, the reproduction ability of the pea aphids was positively proportional to its protein content: the greater protein content, the higher the reproduction ability (Raikhel and Dhadialla, 1992). The protein content of *Nilaparvata lugens* declined under elevated CO_2_, speculated that it would be difficult for it to obtain nutrients and energy after feeding on rice which grown under high CO_2_ concentrations (Zeng et al., 2012). Similarly, elevated CO_2_ decreased the protein content of *Helicoverpa armigera* larvae by 14.16% (Wu et al., 2006). Therefore, we propose that the elevated CO_2_ inhibited protein synthesis in the aphids, and directly led to the reduction of protein contents, thus further caused the longer nymph duration and lower female fecundity of the pea aphids in elevated CO_2_ levels than those of the aphids under the ambient CO_2_.

The total lipid and soluble sugar contents were significantly increased by the elevated CO_2_ levels at F_0_ generation (Table 2). Interestingly, over the generations, they were not significantly changed under the elevated levels of CO_2_, neither generation nor its interaction with CO_2_ level had a significant effect on them (Table 2 and Supplementary Figures 2B, 3B). However, the total lipid and soluble sugar contents were significantly higher under the elevated CO_2_ levels than those under the ambient CO_2_ condition at each generation (Table 2 and Supplementary Figures 2A, 3A). The function of soluble sugar is to offer nutrition for the energy requirement of the muscles when an insect is walking or escaping (Hansford and Johnson, 1975). These increased responses to elevated CO_2_ could be the initiation for their escaping from unsuitable environment. Our study results show that glycogen content increased with the elevated CO_2_ concentration in the pea aphid, which could reduce the feeding rate of the aphids, thus longer nymph duration and reduced adult fecundity under elevated CO_2_ than that under the ambient CO_2_.

On other hands, elevated CO_2_ can lead to the changes of plant secondary metabolism, thus affect the insect physiology indirectly (Bidart-Bouzat and Imeth-Nathaniel, 2008; Todgham and Stillman, 2013). The soluble sugars in the aphid body are mainly from the sap of the phloem of the host plant (Li et al., 2008). Elevated CO_2_ could enhance plant biomass and leaf area, so soluble sugar levels increase, leading to enhance the behavior of the pea aphids (Curtis and Wang, 1998). The reduced metabolic efficiency of plants under elevated CO_2_ could weaken the feeding of insect herbivores and slow the growth of insects (Vuorinen et al., 2004). Elevated CO_2_ could also increase the concentrations of various phenolic compounds, which would act to reduce the pest population and affect natural enemies (Koricheva et al., 1998). For example, the content of flavone, phenolics and condensed tannins and gossypol were higher in the aphid host plant the alfalfa, *Medicago sativa* (L.) grown under elevated CO_2_ (Sun et al., 2019). Carbohydrate content in plants increased with elevated atmospheric CO_2_ due to higher photosynthetic rates (Reddy et al., 2010; Kimball, 2016). Elevated CO_2_ increased carbon to nitrogen ratio of plant tissues (Wilsey, 1996). This causes lower nitrogen concentrations in plants and leads to less amino acids available to aphids (Guo et al., 2013b; Ziska et al., 2016). It is widely recognized that the reproductive ability and abundance of aphids are largely determined by the availability of amino acids in their diet (Jansson and Smilowitz, 1986), and the nutritional quality and resistance of host plants (Guo et al., 2014).

In the current study, the significant effects of the elevated CO_2_ on the growth of the aphids fed with alfalfa leaves were studied for over 6 generations, which is longer than previous studies of 3 generations. Our study clearly demonstrates that the elevated CO_2_ affect some parameters such as nymph developmental duration after 3 generations and shows that the final consequence of the elevated CO_2_ on aphid population dynamics is combinatory and long time. The results will guide further field experiments to evaluate the effects of the elevated CO_2_ conditions on the development of the pea aphids and other insects under climate change conditions.

## Data Availability Statement

The original contributions presented in the study are included in the article/Supplementary Material, further inquiries can be directed to the corresponding author/s.

## Ethics Statement

Informed consent was obtained from all individual participants included in the study. The research project was conducted on invertebrate species that are not subjected to any specific ethical issue and legislation.

## Author Contributions

CuL, QS, and CaL conceived and designed research. CuL, QS, and QZ conducted the experiments. YG and KZ contributed new reagents and analytical tools. CuL and J-JZ analyzed the data. CuL and CaL wrote the manuscript. J-JZ made critical revision, proofreading, and replying comments. All authors read and approved manuscript.

## Conflict of Interest

The authors declare that the research was conducted in the absence of any commercial or financial relationships that could be construed as a potential conflict of interest.
