# Supplementary figures and images for: Long-Term Effect of Elevated CO2 on the Development and Nutrition Contents of the Pea Aphid (Acyrthosiphon pisum)
Source: Front Physiol. 2021 Jun 4;12:688220. doi: 10.3389/fphys.2021.688220 (PMC8213344; doi:10.3389/fphys.2021.688220)

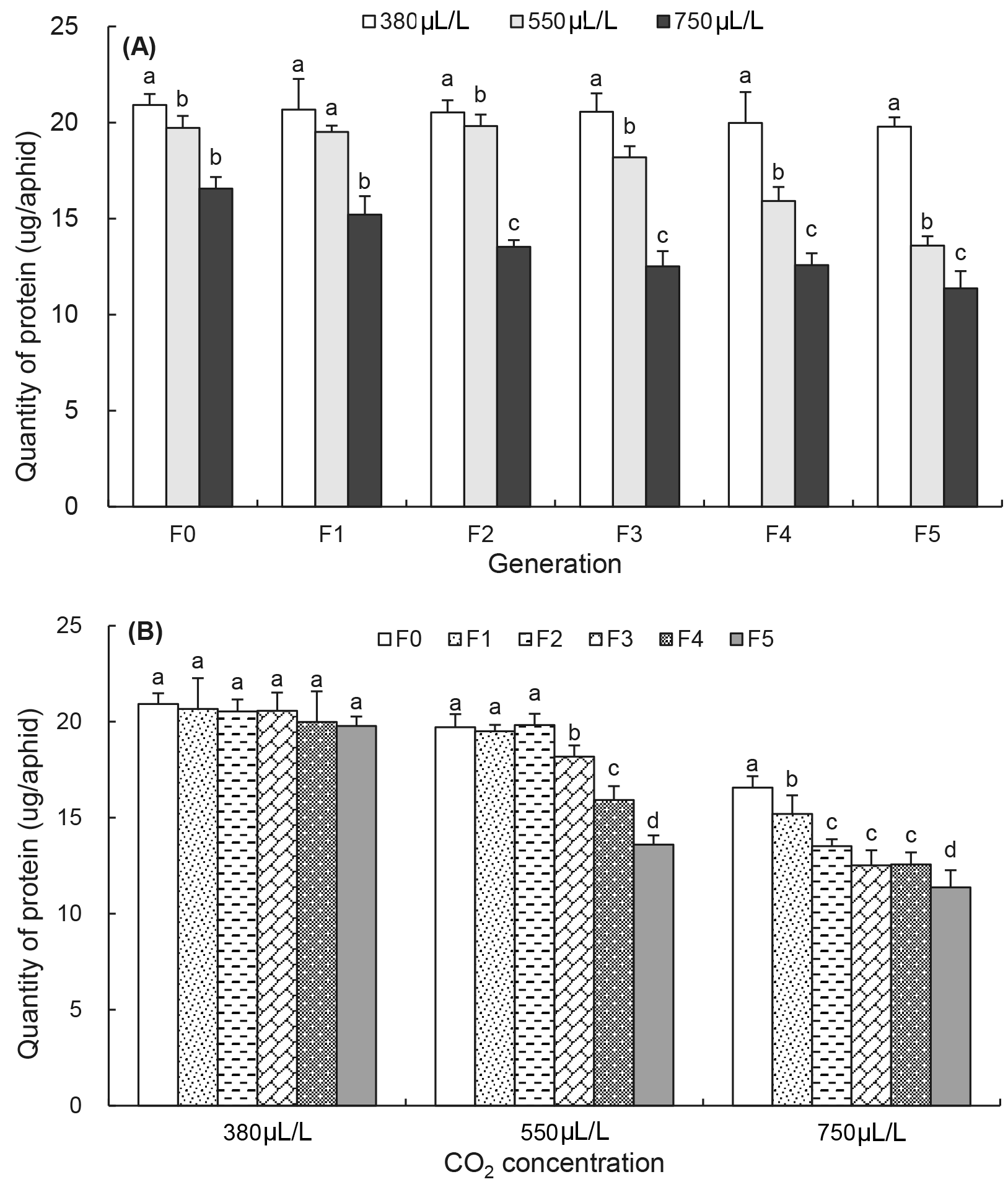

Supplement: Supplementary file 1 [file Image_1.TIF]

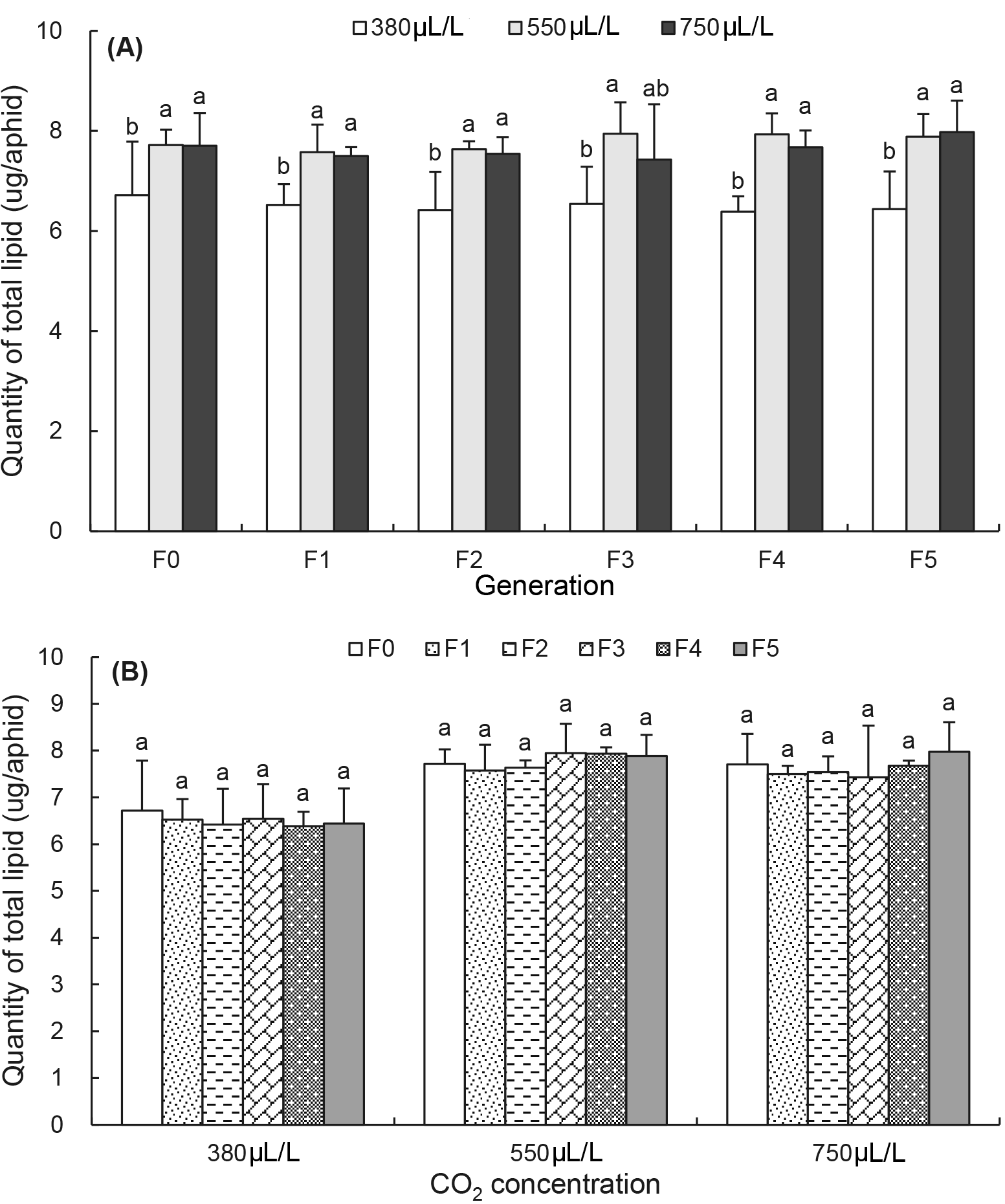

Supplement: Supplementary file 2 [file Image_2.TIF]

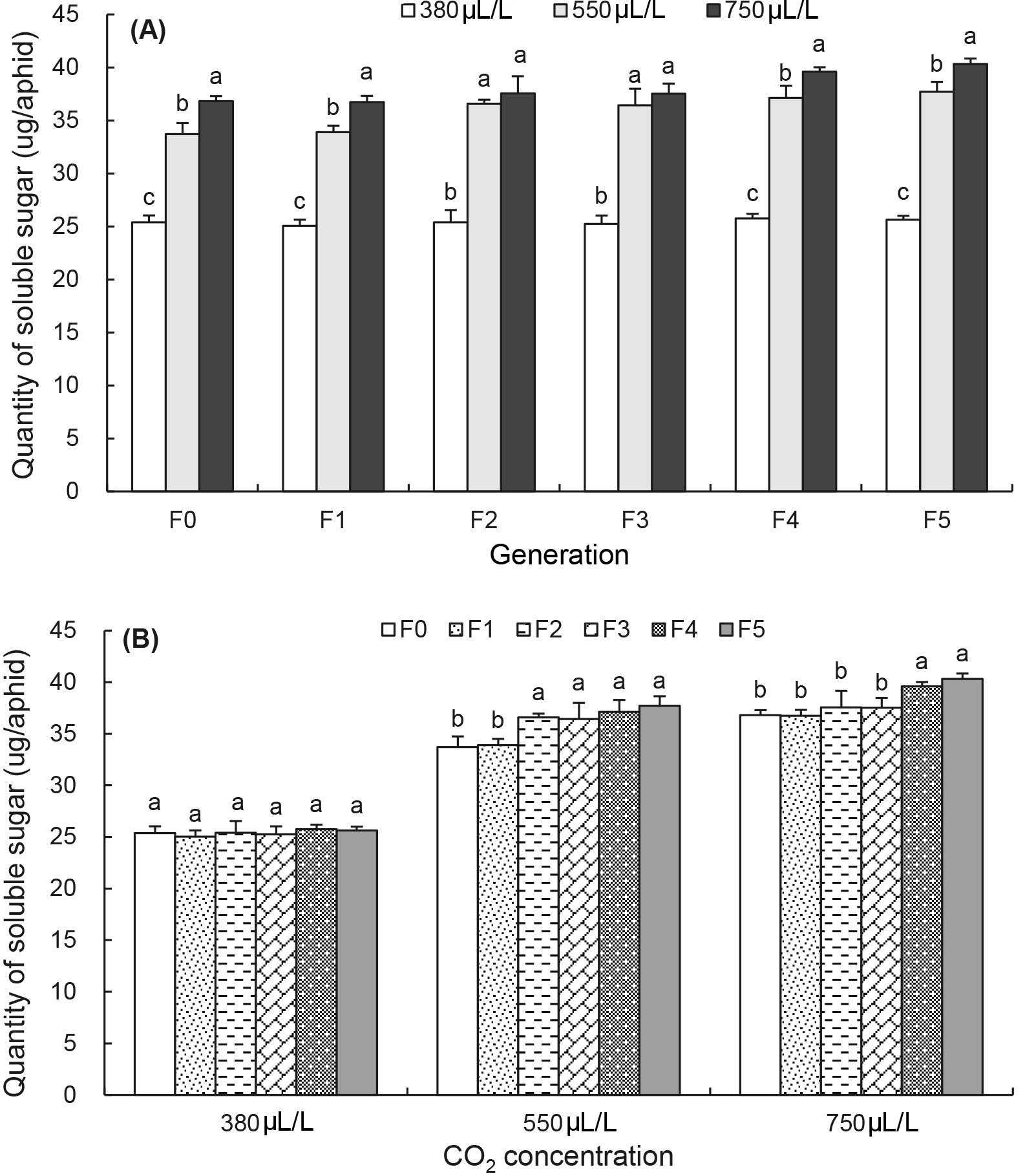

Supplement: Supplementary file 3 [file Image_3.TIF]

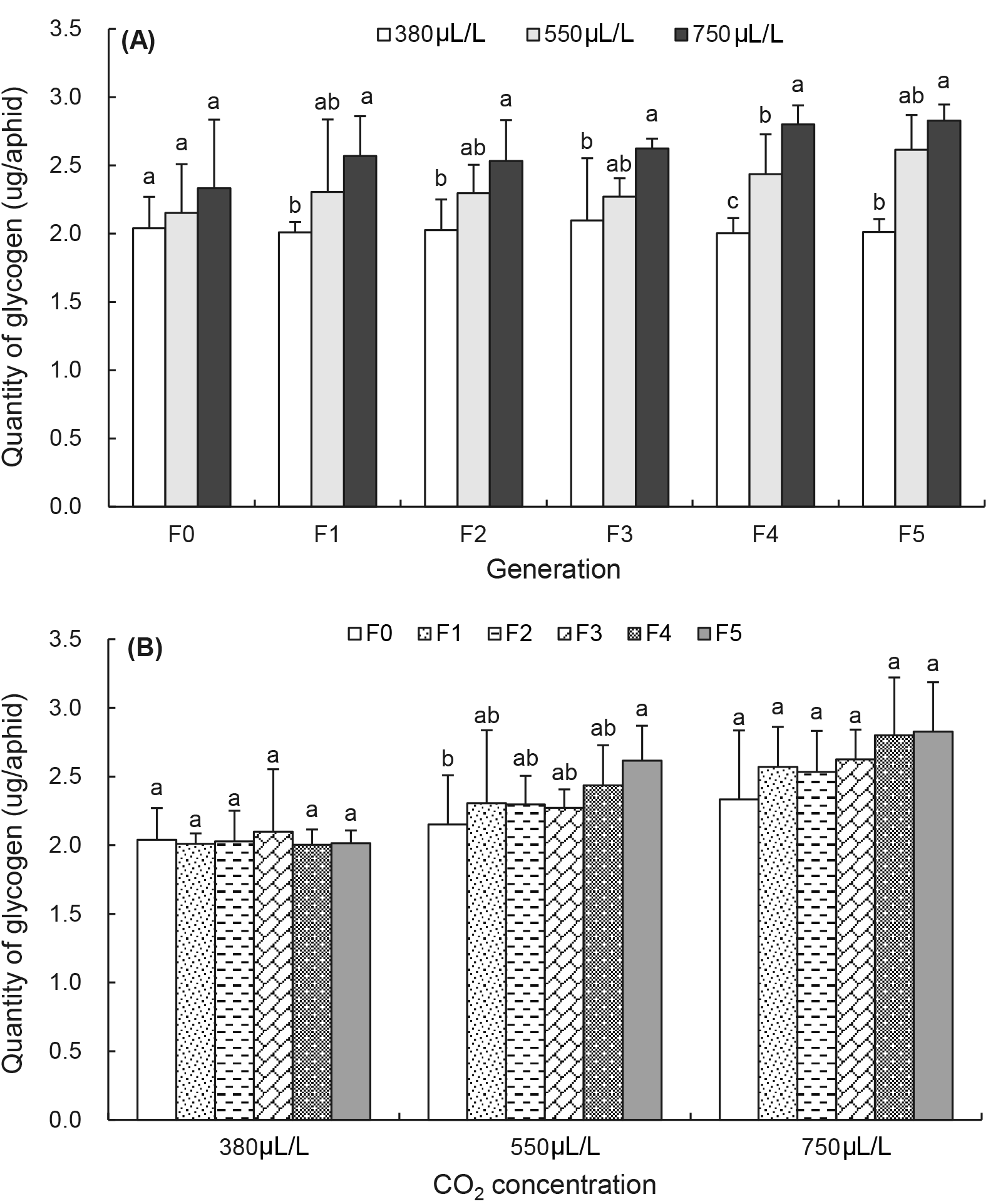

Supplement: Supplementary file 4 [file Image_4.TIF]
